# A Microvalve Module with High Chemical Inertness and Embedded Flow Heating for Microscale Gas Chromatography

**DOI:** 10.3390/s21020632

**Published:** 2021-01-18

**Authors:** Hsueh-Tsung Lu, Yutao Qin, Yogesh Gianchandani

**Affiliations:** 1 Center for Wireless Integrated MicroSensing and Systems (WIMS^2^), University of Michigan, Ann Arbor, MI 48109, USA; yutaoqin@umich.edu (Y.Q.); yogesh@umich.edu (Y.G.); 2 Department of Mechanical Engineering, University of Michigan, Ann Arbor, MI 48109, USA; 3 Department of Electrical Engineering and Computer Science, University of Michigan, Ann Arbor, MI 48109, USA

**Keywords:** solenoid, phosphonates, organophosphorus compounds, chemical warfare agents, volatile organic compounds

## Abstract

This paper reports a multi-valve module with high chemical inertness and embedded flow heating for microscale gas chromatography (µGC) systems. The multi-valve module incorporates a monolithically microfabricated die stack, polyimide valve membranes, and solenoid actuators. The design incorporates three valves within a single module of volume 30.2 cm^3^, which is suitable for the small form factor of µGC systems. The die stack uses fused silica wafers and polyimide valve membranes that enhance chemical inertness. The monolithic die stack requires only three lithographic masks to pattern fluidic microchannels, valve seats, and thin-film metal heaters and thermistors. The performance of fabricated multi-valve modules is compared to a commercial valve in tests using multiple volatile organic compounds, including alkanes, alcohols, ketones, aromatic hydrocarbons, and phosphonates. The valves show almost no distortion of chromatographic peaks. The experimentally measured ratio of flow conductance is 3.46 × 10^3^, with 4.15 sccm/kPa in the open state and 0.0012 sccm/kPa in the closed state. The response time is <120 ms.

## 1. Introduction

### 1.1. Motivation and Need

Microvalves are of great interest for many microsystem applications, such as microscale gas chromatography (μGC), cooling and refrigeration, fuel cells, lab-on-a-chip, and drug delivery [1,2,3,4,5,6,7,8]. The general requirements of microvalves include high flow modulation capability, low leakage, low power consumption, and manufacturability. The microsystem applications that are related to μGC, which is relevant to industrial pollution monitoring, breath analysis, and homeland security, impose additional requirements. These include: (1) low dead volume to reduce chromatographic peak distortion, (2) superior chemical resistance to prevent analyte loss, (3) embedded flow heating in order to reduce surface adsorption of the analytes, and (4) monolithic integration of multiple valves to reduce the overall device volume.

Chemical inertness and embedded flow heating are particularly important for µGC valves that are used in order to analyze organophosphorus compounds. These compounds pose technical challenges to µGC systems, because they are extremely surface-adsorptive and easily retained by the chemically active sites or cold spots on the device inner surfaces [9]. Certain organophosphorus compounds have high toxicity that can disrupt the nerve system, cause respiratory paralysis, and inflict death [10]; these are used in chemical warfare agents (CWAs). Among the different classes of CWAs, nerve agents, such as sarin, soman, and tabun, are one of the most lethal. For general research, simulants, such as dimethyl methyl phosphonate (DMMP) and diethyl methyl phosphonate (DEMP), are commonly used [11]. These possess similar molecular structures, volatilities, and chemical properties but are less hazardous.

Despite an evident need, previously reported µGC systems have not incorporated microfabricated valves [12,13,14,15,16,17,18,19]. Whereas some µGC systems avoided the use of valves entirely [20,21,22,23,24], many others used commercial off-the-shelf (COTS) valves that were not microfabricated. In the latter case, most of the µGC systems avoided using the valves in the fluidic path of sample injection, separation, and detection. These µGC systems typically required multiple valves for controlling the gas flow. In some µGC systems, where upstream flow modulation was inevitable, it was not directly provided by valves, but by a Deans switch [16,17]. The flow routing in these µGC systems would have benefited from the availability of valve with high chemical inertness, low dead volume, and embedded flow heating capability, preferably in a small form factor combining multiple valves in a single package.

### 1.2. Current State-of-Art

Microvalves can generally be categorized based on the actuation method [25]. Electrostatic actuation provides fast responses, low power consumption, and the potential for large-scale integration [26,27,28,29,30,31]. Nonetheless, the displacement that is generated by electrostatic actuators is on the order of a few microns, which creates a large flow resistance in the open state. Piezoelectric actuation provides fast response with low power consumption [32,33,34,35,36]. Two types of piezoelectric actuators are commonly used: lead zirconate titanium (PZT) stacks and piezoelectric bimorph disk actuators. PZT stacks typically provide large blocking force (>100 N), but small displacement (<10 µm), whereas piezoelectric bimorph disk actuators generate large displacement (50–500 µm) and moderate blocking force (<1 N). Thermopneumatic actuation provides large displacement and high blocking force, but it requires high power consumption and provides slow response times [37]. A hybrid actuation scheme combining thermopneumatic actuation with electrostatic actuation has been reported to reduce the power consumption in valves that are intended for µGC systems [38]. Solenoid actuation is the most common kind in commercial miniature valves; it offers both high blocking force and large displacement [39,40,41]. The blocking force and displacement of solenoid actuators are easily customizable. Although solenoid actuation requires relatively high peak power (8–20 W), latching solenoid actuators consume power only while switching.

Other than the actuator, the general structure of microvalves includes fluidic channels, thin suspended valve membranes, and valve seats, which are typically microfabricated on silicon, glass, or silicon-on-insulator substrates [1,2,3,4,8,26,27,28,32,33,34,35,36,38,39,40]. The wetted materials include glass, silicon, and inorganic dielectric materials, which are chemically inert to many volatile organic compounds (VOCs). However, integrated membranes made of these materials can present challenges with complex fabrication, fragility, and low tolerance to dust particles. In other microvalves, the valve membranes are made of polytetrafluoroethylene [42] or polyimide [36], which reduce the fabrication complexity. However, the coupling to external actuators and the alignment during assembly remain challenging.

**Figure 1:**
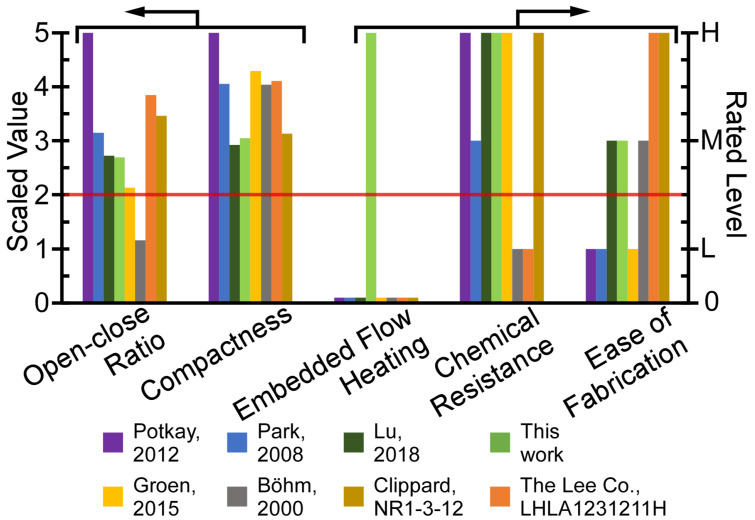
A multi-dimensional benchmarking of microvalves in the context of microscale gas chromatography (µGC). The minimum desired level for µGC is indicated by the red line. The microvalve module in this work uniquely provides both embedded flow heating capability and superior chemical resistance, while only modestly compromising other metrics.

The internal structure of some COTS valves is similar to microfabricated valves; it includes a solenoid actuator, fluidic paths, and a valve membrane (e.g., NIV series from Clippard, Cincinnati, OH, USA, 075T series from Bio-Chem Fluidics, Inc., Boonton, NJ, USA, and LFN series from The Lee. Co., Westbrook, CT, USA). Some other COTS valves do not use a valve membrane, but instead use an elastomer plunger head that is directly attached to the actuator (e.g., LHL series from The Lee. Co., Westbrook, CT, USA); this approach can present chemical compatibility challenges and also increase dead volume. Microfabricated and COTS valves have both historically lacked embedded flow heaters, presenting challenges for the analysis of heavy and surface-adsorptive chemicals in µGC systems, such as organophosphorus compounds. In order to compare the valves within the context of µGC, the performance can be summarized in five metrics: open-close ratio of flow conductance, ease of fabrication, compactness (which is inversely related to size), chemical resistance, and embedded flow heating. For most µGC systems and other systems where frequent switching of valve is not required, the power consumption and response time are less critical, and, therefore, not compared. In Figure 1, two metrics—open-close ratio of flow conductance, and compactness—that can both be quantitatively evaluated are scaled to a range of 0–5; three other metrics—embedded flow heating, chemical resistance, and ease of fabrication—which can only be qualitatively evaluated, are rated high, medium, or low. The open-close ratio of flow conductance is evaluated based on the actual performance; the compactness is evaluated based on the device volume; ease-of-fabrication is estimated from the lithographic mask count, except for the COTS valve; and, chemical resistance is estimated from the wetted materials. For µGC systems, the microvalve should preferably merit at least a moderate score in each of the five metrics.

This paper reports compact microvalves that incorporate the necessary features of high chemical inertness, embedded flow heating capability, high open-close ratio, and ease of fabrication. Multiple microvalves are incorporated within a module that is comprised of a chemically inert microfabricated fluidic die stack with an embedded thin-film heater, polyimide valve membranes, and solenoid actuators that are placed within a 3D printed housing. The module is intended for a new type of µGC system [43] that requires up-stream flow routing between a sampling path and multiple separation paths and heavily depends on a high performance microvalve module. The module structure and operation are described in Section 2, the computational modeling results in Section 3, the experimental results in Section 4, and the conclusion in Section 5.

## 2. Device Structure and Operation

### 2.1. Device Structure and Fabrication

**Figure 2:**
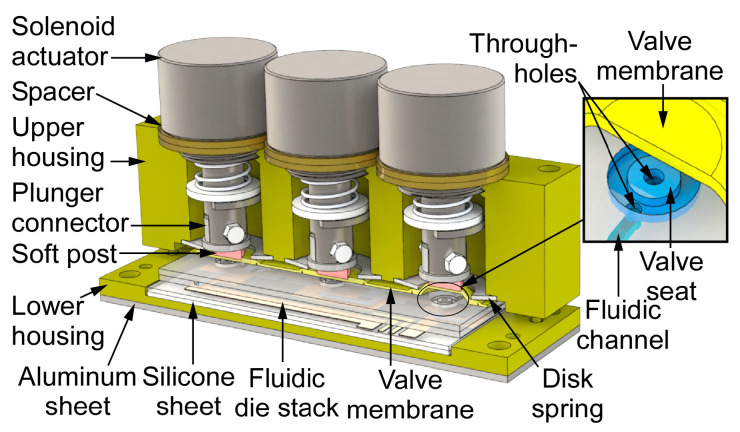
The architecture of the microvalve module. The microvalve on the right is in the open state, whereas the other two of the microvalves are in the closed state.

**Figure 3:**
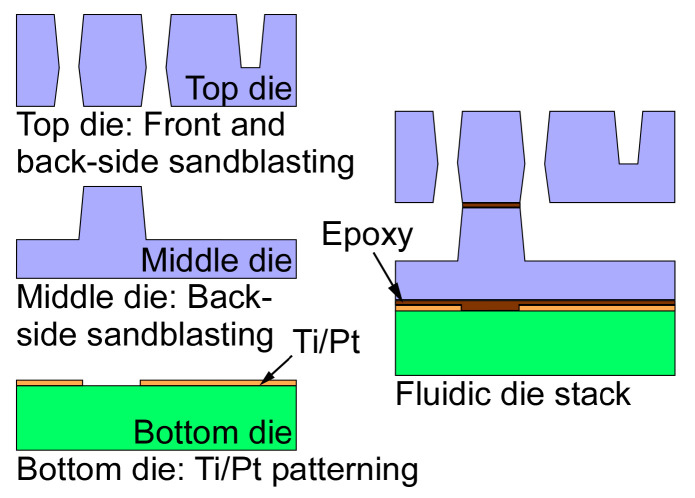
The die stack fabrication process. The valve seats, fluidic microchannels, and through-holes are created by a two-mask sandblasting process. The Ti/Pt layer is patterned while using another mask. Three dies are bonded into a stack to form the fluidic die stack.

**Figure 4:**
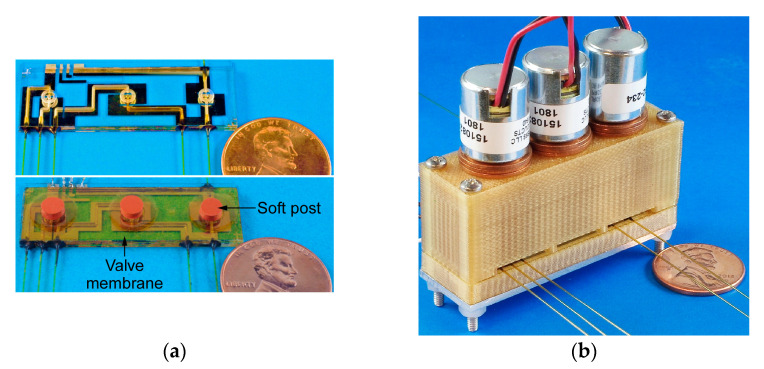
(**a**) Photographs of the fluidic die stack prior to and following the assembly of the valve membrane and soft post. (**b**) Final view of the fully assembled microvalve module.

The microvalve module integrates three valves, which incorporate a monolithically microfabricated fluidic die stack, prefabricated valve membranes, solenoid actuators, and a customized three-dimensional (3D) printed housing (Figure 2). The fluidic die stack is made of fused silica, which contains lower metal content and, hence, provides higher chemical inertness than glass; the stack includes three microfabricated dies. The top die comprises valve seats and through-holes; each valve seat separates two through-holes (Figure 2 inset) and it is designed in a ring shape in order to avoid corners that may create dead volume. The through-holes connect the fluidic paths of the top die to the middle die, which routes the flow to ports that are located on its perimeter. The ports are 600 µm wide and 450 µm deep for accommodating capillary tubing. All of the microchannels are 400 µm wide and 425 µm deep, with a sidewall taper of 22° off vertical. The total microchannel length of the three valves in each module ranges from 1.1 cm to 3.7 cm. The valve seats and fluidic microchannels on the top die and the middle die are created by two-sided sandblasting of a 700 µm thick fused silica wafer. The bottom die, which is comprised of a 500 µm fused silica substrate, incorporates a thin-film thermistor and a joule heater that follows the fluidic microchannels on the middle die; both the thermistor and heater are made of Ti/Pt. The three dies are bonded together by epoxy (Epotek 377, Epoxy Technology, Inc., Billerica, MA, USA) (Figure 3) into a die stack that is 3.9 × 1.4 × 0.2 cm^3^ (Figure 4a).

An ideal valve membrane must provide a chemically inert surface, leak-free mating with the valve seat, flexibility for low force actuation, and infrangibility for assembly and repeated operations. A membrane that is sufficiently flexible can also accommodate the presence of dust particles. In this work, a two-layer polyimide membrane is used as the valve membrane. Each layer is a self-adhesive polyimide film with adhesive on one side. The lower layer has a circular shape and it is positioned above the valve seat with the adhesive side facing up, whereas the upper layer has a larger size and rectangular shape with the adhesive side facing down. Thus, the upper layer holds the lower layer on the valve seat and seals the perimeter of each valve seat. The three valves share one upper layer to simplify assembly. A disk spring at each of the three perimeters secures the membrane. With this structure, the membrane adhesive is not exposed to the flow path; the wetted materials are polyimide and fused silica, which are chemically inert to VOCs of interest in this work.

Each microvalve is actuated by a solenoid actuator (151082-234, Johnson Electric, Shatin, NT, Hong Kong), which consists of a magnet, an electromagnetic coil, and a spring. Each actuator is mechanically coupled to the valve membrane through a plunger that ends in a post of adhesive coated soft silicone or adhesive foam (VHB 5962, 3M, Saint Paul, MN, USA). The plunger may be aluminum or 3D printed Ultem1010 polymer. This approach provides a conformable seal against the valve seat.

A two-part housing is used in order to accommodate the components with precise alignment. It is 3D printed from Ultem1010^®^ (CIdeas Inc., Crystal Lake, IL, USA) because of the high printing resolution and thermal stability. The upper housing incorporates multiple chambers for the solenoid actuators and disk springs and jigs for alignment to the lower housing, which incorporates a cavity for the fluidic die stack and alignment holes. Nylon spacers are used to adjust the vertical position of the actuators and, consequently, the actuator displacement. The fluidic die stack is seated within the cavity on a 500 µm-thick silicone cushion to absorb shock. In addition, a 1.5 mm-thick aluminum sheet is attached beneath the lower housing for mechanical rigidity and robustness. Screws and nuts are used to fasten the upper and lower housing. The size of the assembled device is 5.3 × 1.7 × 4.5 cm^3^ (Figure 4b).

### 2.2. Operation

**Figure 5:**
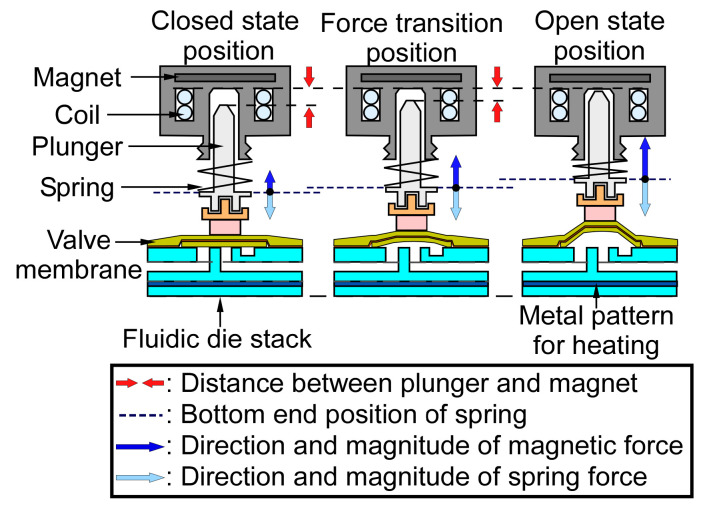
The relationship between the distance and the force of the solenoid actuator. The closed state position has a net force exerting downward, whereas the open state position has a net force exerting upward.

The operation of the microvalve can be explained by the separation between the plunger and the latching magnet, and by the compression of the spring (Figure 5). When the microvalve is in its closed state, the spring is moderately compressed, and it maintains a spring force against the fluidic die stack. When the solenoid actuates the plunger, pulling it toward the magnet, the compression of the spring increases, but the attractive force from the latching magnet increases more rapidly. At the force transition position, the spring force and magnetic force are balanced. When the microvalve is in its open state, the magnetic force dominates over the spring force, and it latches the microvalve, even when the solenoid is turned off. To open the microvalve from the closed state, a positive 12 V, 0.9 A pulse is applied to the solenoid for 25 ms. A negative 12 V, 0.9 A pulse is applied for 25 ms in order to close the microvalve from the open state. The spring (CCF-0600-0013-M, The D.R. Templeman Co., Plainville, CT, USA) provides a displacement of 560 µm and a blocking force of 2.8 N.

## 3. Modeling Results

The flow resistance of the microvalve in the open state was estimated by finite element analysis (FEA) while using COMSOL Multiphysics software. The simulated structure represented the gas flow path of the middle valve in Figure 1, i.e., it included two microchannels, the valve seat, and the valve membrane region. The height of the microchannels was assumed to be 425 µm, and the sidewall taper was represented. The height of the valve membrane region, which is determined by the actuator displacement, was assumed to be 560 µm.

**Figure 6:**
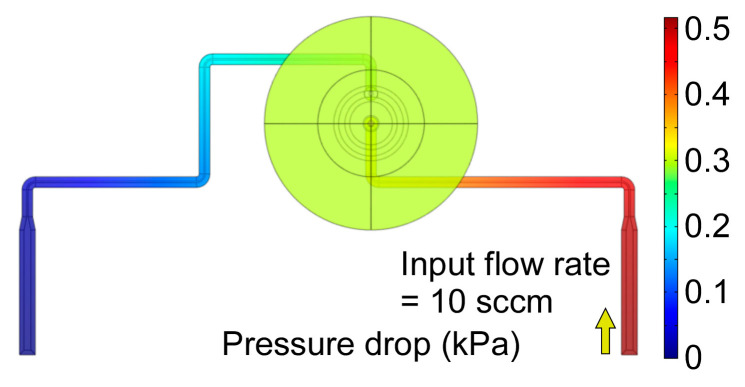
The pressure distribution along the fluidic path of the middle valve of the microvalve module.

The simulation assumed laminar flow. A flow rate of 10 sccm was supplied to the inlet, while the outlet was assumed to be at ambient pressure. The simulated pressure drop across the microvalve was 0.52 kPa (Figure 6), with a pressure drop of ≈0.21 kPa in the fluidic microchannel upstream of the valve membrane region, ≈0.02 kPa in the valve membrane region, and ≈0.29 kPa in the fluidic microchannel downstream of the valve membrane region. The overall flow resistance of the microvalve in the open state was 0.052 kPa/sccm. As evident from the pressure distribution, the flow resistance of the membrane region was negligible because of the large membrane displacement.

**Figure 7:**
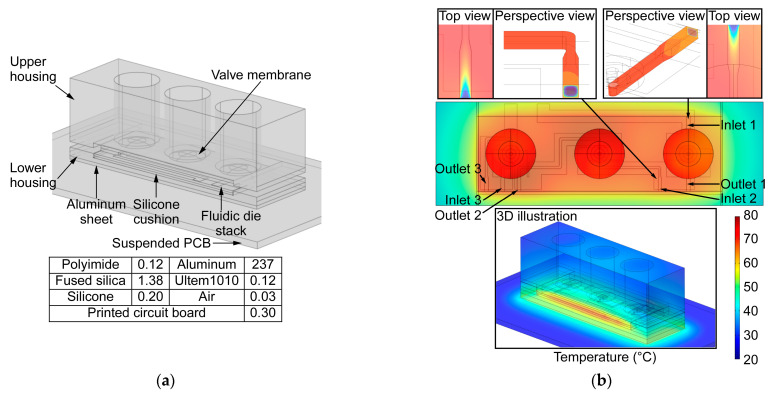
(**a**) The geometric model and the thermal conductivity values (Wm^−1^K^−1^) used in the simulation. (**b**) The temperature distribution of fluidic die stack with a simulated heater power of 1.1 W. Close-up views of the valve inlets are shown in the insets.

The thin-film heater and thermistor allow the microvalve to be compatible with surface-adsorptive chemical species. The thermal characteristics of the valve were simulated by FEA while using COMSOL Multiphysics software. The simulated structure (Figure 7a) included the entire lower housing and its contents, as well as the upper housing; for simplicity, the simulated structure excluded the actuators, plunger connectors, and soft posts, all of which contributed to negligible heat conduction from the heated fluidic die stack because of the low thermal conductance and small contact area of the soft posts. The empty space in the microvalve module was modeled as air. Additionally, the microvalve module was assumed to be located on a suspended printed circuit board (PCB), thus allowing natural air convection on all of the exterior surfaces. The convective heat transfer coefficients were computed within COMSOL based on the configuration and characteristic lengths of the exterior surfaces.

The three valves were heated up simultaneously, as they shared one embedded heater. With heater power of 1.1 W, the temperature around the thermistor area (*T_therm_*) rose from room temperature (*T_rm_*: 23 °C) to 75 °C, whereas the lowest temperature in the fluidic paths rose from *T_rm_* to ≈65 °C (Figure 7b). Each valve was assumed to have 10 sccm air flow rate at its inlet to mimic a µGC system in sampling mode. The temperature of the incoming flow rose from *T_rm_* to 90% of steady-state temperature within a travel distance of 1.9 mm past the inlet. The resulting temperature distributions for the middle and right valves are shown in the insets of Figure 7b. This simulation confirmed that the thin-film heater can provide adequate heating for analyzing heavy chemical species without consuming excessive power. It also showed that, even with high air flow supplied to the microvalve inlet, the incoming sample stream can be rapidly heated.

## 4. Experimental Results

### 4.1. Flow Conductance Tests

**Figure 8:**
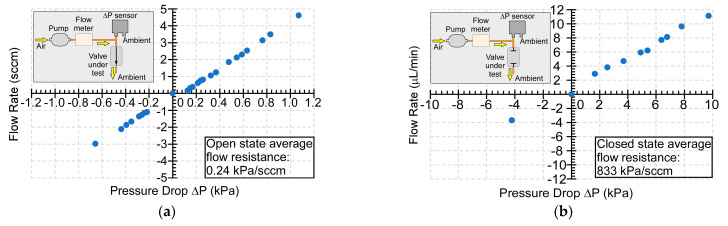
(**a**) Flow characteristics for an open microvalve. (**b**) Leakage characteristics for a closed microvalve. The test setup for each case is shown in the insets.

The flow conductance of the assemble device was evaluated while using a setup that consisted of a pump (mp6, Bartels Mikrotechnik GmbH, Germany), a differential pressure sensor (MPXV5010DP, NXP, Austin, TX, USA), and flow meters (FMA 1603A & 1601A, Omega Engineering, Inc., Norwalk, CT, USA) (Figure 8 insets). The flow rates through the microvalve and the corresponding pressure drop across the microvalve were measured while varying the blocking pressure of the COTS pump module.

The tests were conducted at room temperature while using ambient air as the flow medium. Air was supplied by the pump in one of two regimes of microvalve operation: positive pressure or negative pressure. In the positive pressure regime, the pressure within the valve membrane region was higher than the ambient pressure. This generated a force pushing against the valve membrane and counteracting the latching force in the closed state. In the negative pressure regime, the pressure within the valve membrane region was lower than the ambient pressure. Thus, this generated a force pulling down the valve membrane and counteracting the latching force in the open state.

In the open state, the measured flow rates increased proportionally with the measured pressure drops across the microvalve over a range of −0.66 kPa to 1.07 kPa (Figure 8a). The slope of relationship between the flow rate and pressure drop indicated that the microvalve flow conductance was 4.15 sccm/kPa when open. When the microvalve was closed, the measured flow rate showed a linear relationship with the measured pressure drop over a range of −4.21 kPa to 9.72 kPa (Figure 8b), with a leakage conductance of 0.0012 sccm/kPa. Based on these results, the typical open-close ratio of flow conductance of the microvalve was 3.46 × 10^3^. The microvalve showed normal operation in both the positive pressure and negative pressure regimes. The microvalve could withstand positive pressure of 9.72 kPa in the closed state and negative pressure of 0.66 kPa in the open state, respectively. Higher magnitudes of negative pressure were not tested, because the resulting flow rates would exceed the need of typical µGC systems.

The flow conductance was measured with two capillary tubes that were attached to the two gas flow ports of the microvalve. The two capillary tubes had an inner diameter of 250 µm and a total length of 6 cm. Estimated by the Poiseuille’s law [44], the two capillary tubes presented a total flow resistance of 0.187 kPa/sccm. After subtracting the flow resistance of capillary tubes from the experimental flow resistance, the flow resistance of the microvalve was determined as 0.053 kPa/sccm, being only 1.9% different from the simulated flow resistance (i.e., 0.052 kPa/sccm).

### 4.2. Dynamic Response Test

**Figure 9:**
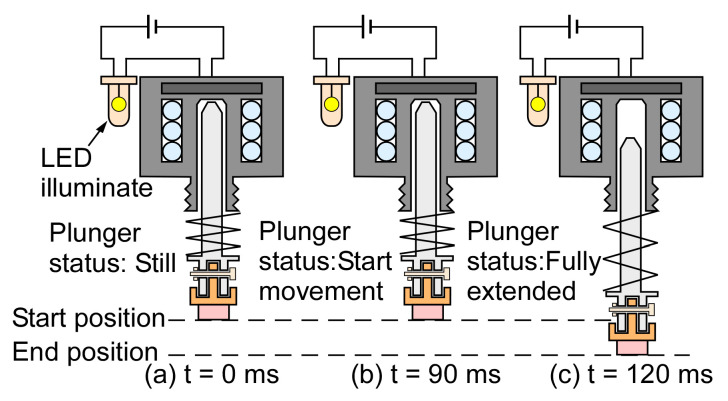
Dynamic response of the microvalve. (**a**) At 0 ms, the light emitting diode was turned on, confirming the passage of electric current. (**b**) At 90 ms, the plunger started to move. (**c**) At 120 ms, the plunger reached the end position.

To study the dynamic response of the actuator, the actuator operation was recorded by a video camera. A light emitting diode (LED) was connected with the solenoid actuator as an indicator of the applied current. At time 0, the current started to illuminate the LED; at time 90 ms, the plunger started to move; at time 120 ms, the plunger reached the end position (Figure 9). These measurements indicated that there was 90 ms delay between the application of the actuation current and the start of actuation, and 30 ms duration for the actuation. Therefore, the overall time that was required for a complete actuation was ≈120 ms.

### 4.3. Power and Energy Consumption

**Figure 10:**
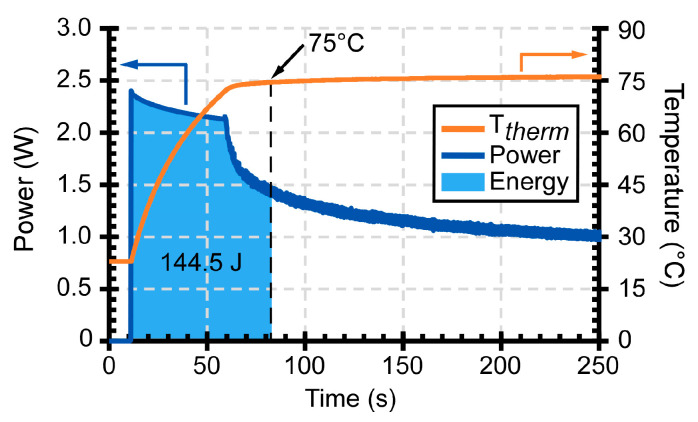
The energy and power consumption of the microvalve module during heating. The microvalve was heated to and maintained at 75 °C under servo control.

During the switching transients, the actuator consumed ≈0.27 J per switch. In the latched states (i.e., fully open and fully closed states), power was only consumed for the flow path heater, if any. The embedded flow heating was controlled by a close-loop algorithm in LabVIEW, in which the input current and voltage were monitored. The microvalve required ≈144.5 J to be heated to 75 °C; this temperature could be maintained with an input power of ≈1.0 W (Figure 10). The measured result matched the simplified thermal modeling result with only 10% difference. The maximum power that was provided in this test was limited to ≈2.4 W, although higher power could be used to accelerate the heating.

### 4.4. Lifetime Test

**Figure 11:**
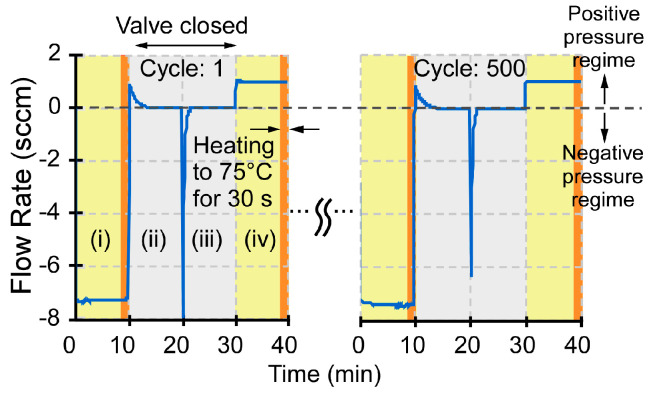
Cycling lifetime test of the microvalve. The sudden change of flow rate at t = 20 min. resulted from an imposed reversal of flow direction.

Extended lifetime was tested by cyclical operation. During each testing cycle, the microvalve was tested with bidirectional flow in open and closed states. The testing conditions included four stages: (i) microvalve open in the negative pressure regime, (ii) microvalve closed in the positive pressure regime, (iii) microvalve closed in the negative pressure regime, and (iv) microvalve open in the positive pressure regime. In the last 30 s of the open state, i.e., stage (ii) and (iv), the microvalve was heated to 75 °C (Figure 11). The tests revealed a lifetime >500 cycles over a duration of 330 h without any degradation in performance. These results were obtained for the microvalve version that used an aluminum plunger that was attached to the valve membrane with adhesive foam. The microvalve version that used a silicone post provided lower lifetime when heated, because of the failure of the adhesive that was used to bond silicone post to the valve membrane under applied stresses.

### 4.5. Chemical Injection Tests

**Figure 12:**
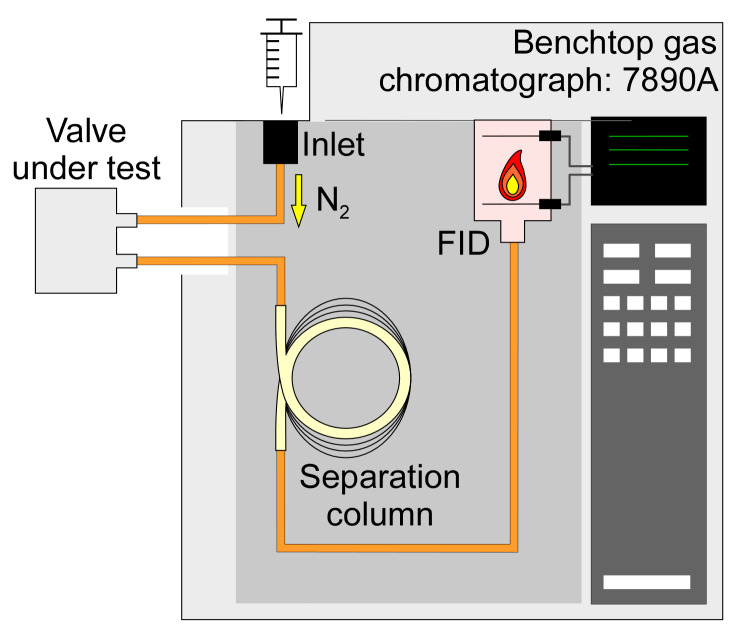
The test setup of the chemical injection tests. The valve under test was connected to the flow path of a benchtop gas chromatograph (7890A, Agilent Technologies Inc., Santa Clara, CA, USA).

The impact of the valve on chromatographic peaks was evaluated while using a benchtop gas chromatograph (7890A, Agilent Technologies, Inc., Santa Clara, CA, USA). This instrument was equipped with an oven, within which were located a flame ionization detector (FID), and a separation column with 1 m length, 320 µm inner diameter, and 5 µm thick OV-1 stationary phase coating (115-3206, Ohio Valley Specialty Company, Marietta, OH, USA) (Figure 12). The valve under test was located outside the oven and connected along the flow path, downstream of the inlet and upstream of the separation column and the FID. The column was maintained at 80 °C inside the oven. With 0.6 sccm N_2_ carrier gas flow, the analytes injected at the inlet flowed through the valve in its open state, the separation column, and into the FID.

The experiments included four test cases. In Case 1, a benchmark for the chromatographic peaks was established without any valve; a chemically deactivated capillary load that had equivalent flow resistance to the microvalve was used in place of the valve. In Case 2, the microvalve was operated at 22 °C ambient temperature. In Case 3, the microvalve was operated at 80 °C while using the embedded flow heating. In Case 4, the microvalve was replaced by a COTS valve (LHLA1231211H, The Lee Co., Westbrook, CT, USA) that has been used previously in multiple μGC systems [13,14,15,16,18]. The COTS valve was only operated at 22 °C ambient temperature, as it does not have embedded flow heating capability.

**Figure 13:**
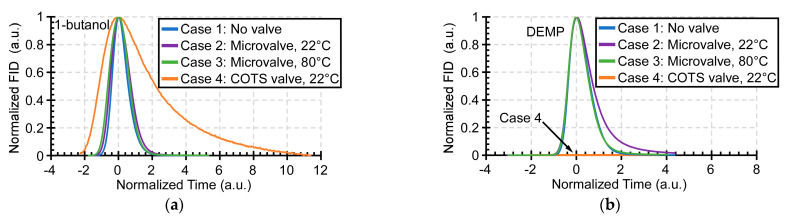
(**a**) Normalized and shifted peaks for 1-butanol. (**b**) Normalized and shifted peaks for diethyl methyl phosphonate.

**Figure 14:**
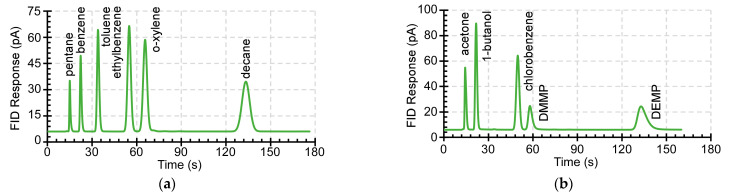
(**a**) The chromatogram of non-polar chemical analytes in Case 3. (**b**) The chromatogram of polar chemical analytes in Case 3.

Two chemicals were selected in order to compare these four cases: 1-butanol, representing light polar species, and DEMP, representing heavy polar species. The former is a polar alcohol with a Kovats retention index of 660, whereas the latter is an organophosphorus compound with a Kovats retention index of 975. The responses of the four cases are compared in the normalized peaks that are presented in Figure 13. The FID signal intensity of each case normalized to its peak is plotted against a time axis. The time axis is centered at the peak and ratioed to peak width at half height (PWHH) for Case 1 in order to compensate for run-to-run variation in the test setup [45]. Thus, a Case that provides a PWHH of 2 on this plot represents a peak that is double the width of the reference Case 1, and the overlay of these normalized peaks readily reveals the chromatogram distortion that is attributable to the valve under test (Figure 14).

For the 1-butanol peak, the unheated microvalve (Case 2) did not cause any noticeable distortion relative to Case 1, but it showed ≈20% broadening for the DEMP peak. The heated microvalve (Case 3) showed no distortion for both the 1-butanol peak and DEMP peak. In contrast, the unheated COTS valve (Case 4) increased PWHH of the 1-butanol peak by ≈240% (Figure 13a). The DEMP peak was virtually eliminated in Case 4, which indicated that DEMP was severely retained by the COTS valve. Thus, the light polar species were slightly affected by the microvalve operating at room temperature, but severely affected by the COTS valve. Further, the heating of the microvalve virtually eliminated distortion. These results were verified with multiple runs of each test case.

Additional chemicals that were tested in Case 3 included a non-polar mixture (containing pentane, benzene, toluene, ethylbenzene, o-xylene, and decane), and a polar mixture (containing acetone, 1-butanol, chlorobenzene, DMMP, and DEMP). In both chromatograms, which are shown in Figure 14, all of the chemical species eluted in the sequence of their expected retention time [45] and did not show significant peak distortion. Overall, the results showed that the heated microvalve does not retain analytes and, consequently, that the sensitivity of µGC systems that incorporate it will not be affected.

## 5. Conclusions

In conclusion, this paper has reported a monolithic multi-valve module that combines microfabricated valve seats, fluidic microchannels, heaters, and thermistors with prefabricated valve membranes and actuators. In addition to the three-valve module, a two-valve module with the same footprint and structure has also been fabricated and tested; it has provided similar results. The integration of microfabricated components and commercially available components has eliminated the need to fabricate thin suspended valve membranes, which simplifies the fabrication and improves the robustness. These features enable the incorporation of the multi-valve modules into µGC systems. The microvalve module has been tested with polar and non-polar species of VOCs and it has demonstrated chromatograms with almost no peak distortion when embedded flow heating is used. The superior chemical resistance and embedded flow heating capability of the microvalve module have also been confirmed as being extremely necessary and critical for µGC; the chemical resistance of the unheated COTS valve has been shown to cause significant peak distortion.

The microvalve has provided a high open-close ratio of flow conductance of 3.46 × 10^3^ and a large flow conductance of 4.15 sccm/kPa (i.e., a flow resistance of 0.24 kPa/sccm) in the open state. It unlike many microfabricated valves, where the low flow conductance was primarily constrained by the membrane displacement that can be generated by the actuators (e.g., [26,28,33,34]), the solenoid actuators that have been used in this work provide a large displacement of 560 μm, and do not limit the flow conductance. This suggests that the flow conductance can be further increased by increasing the hydraulic diameters of the fluidic channels and external capillary tubing. However, the microvalves suffered from minor leakage (0.0012 sccm/kPa) in the closed state. One possible reason is a misalignment between the soft post and actuator, which could cause non-uniform force distribution and, consequently, leave a gap between the valve membrane and valve seat. The use of appropriate alignment jigs and tools in the assembly process may alleviate this issue.

The main power consumption of microvalves resulted from the embedded flow heating. For maintaining the microvalve at 75 °C, as necessary for accommodating certain chemical species, the required embedded flow heating power was ≈1.0 W. The operating temperature can be further increased by providing thermal isolation to the die stack and housing. Additionally, the packaging materials with higher temperature tolerance should be used. These changes will enhance both heating efficiency and heating capability without compromising device lifetime, thereby enabling the device to be used at even higher temperatures in chromatography and sensing systems, such as in certain industrial settings, or in other applications, such as gas fuel delivery.

## Data Availability

Data sharing not applicable.

## References

[1] Terry S.C., Herman J.H., Angell J.B. (1979). A gas chromatographic air analyzer fabricated on a silicon wafer. IEEE Trans. Electron. Devices.

[2] Park J.M., Taylor R.P., Evans A.T., Brosten T.R., Nellis G.F., Klein S.A., Feller J.R., Salerno L., Gianchandani Y.B. (2008). A piezoelectric microvalve for cryogenic applications. J. Micromech. Microeng..

[3] Park J.M., Evans A.T., Rasmussen K., Brosten T.R., Nellis G.F., Klein S.A., Gianchandani Y.B. (2009). A microvalve with integrated sensors and customization normal state for low-temperature environment. J. Microelectromech. Syst..

[4] Yang X., Holke A., Jacobson S.A., Lang J.H., Schmidt M.A., Umans S.D. (2004). An electrostatic, on/off microvalve designed for gas fuel delivery for the MIT microengine. J. Microelectromech. Syst..

[5] Hesketh P.J., Bintoro J.S., Luharuka R. (2004). Microvalve for fuel cells and miniature gas chromatographic system. Sens. Update.

[6] Wang J., Chen Z., Mauk M., Hong K.-S., Li M., Yang S., Bau H.H. (2005). Self-actuated, thermo-responsive hydrogel valves for lab on a chip. Biomed. Microdevices.

[7] Subramani B.G., Selvaganapathy P.R. (2006). A versatile microreactor platform featuring a chemical-resistant microvalve. J. Micromech. Microeng..

[8] Evans A.T., Chiravuri S., Gianchandani Y.B. (2011). A multidrug delivery system using a piezoelectrically actuated silicon valve manifold with embedded sensors. J. Microelectromech. Syst..

[9] Popiel S., Witkiewicz Z., Cazes J. (2009). Chemical warfare agents: GC analysis. Encyclopedia of Chromatography.

[10] Brown M.A., Brix K.A. (1998). Review of health consequences from high-, intermediate- and low-level exposure to organophosphorus nerve agents. J. Appl. Toxicol..

[11] Mitchell M.B., Sheinker V.N., Mintz E.A. (1997). Adsorption and decomposition of dimethyl methylphosphonate on metal oxides. J. Phys. Chem..

[12] Lewis P.R., Manginell R.P., Adkins D.R., Kottenstette R.J., Wheeler D.R., Sokolowski S.S., Trudell D.E., Byrnes J.E., Okandan M., Bauer J.M. (2006). Recent advancements in the gas-phase MicroChemLab. IEEE Sens. J..

[13] Zampolli S., Elmi I., Mancarella F., Betti P., Dalcanale E., Cardinali G.C., Severi M. (2009). Real-time monitoring of sub-ppb concentrations of aromatic volatiles with a MEMS-enabled miniaturized gas-chromatograph. Sens. Actuators B Chem..

[14] Kim S.K., Chang H., Zellers E.T. (2011). Microfabricated gas chromatograph for the selective determination of trichloroethylene vapor at sub-parts-per-billion concentrations in complex mixtures. Anal. Chem..

[15] Garg A., Akbar M., Vejerano E., Narayanan S., Nazhandali L., Marr L.C., Agah M. (2015). Zebra GC: A mini gas chromatography system for trace-level determination of hazardous air pollutants. Sens. Actuators B Chem..

[16] Lee J., Zhou M., Zhu H., Nidetz R., Kurabayashi K., Fan X. (2016). Fully automated portable comprehensive 2-dimensional gas chromatography device. Anal. Chem..

[17] Seeley J.V., Micyus N.J., Bandurski S.V., Seeley S.K., McCurry J.D. (2007). Microfluidic deans switch for comprehensive two-dimensional gas chromatography. Anal. Chem..

[18] Collin W.R., Serrano G., Wright L.K., Chang H., Nunovero N., Zellers E.T. (2014). Microfabricated gas chromatograph for rapid, trace-level determinations of gas-phase explosive marker compounds. Anal. Chem..

[19] Gordenker R.J.M., Wise K.D. A programmable palm-size gas analyzer for use in micro autonomous systems. Proceedings of the SPIE Defense, Security, and Sensing.

[20] Qin Y., Gianchandani Y.B. (2014). iGC1: An integrated fluidic system for gas preconcentrator, column, and detector microfabricated by a three-mask process. J. Microelectromech. Syst..

[21] Qin Y., Gianchandani Y.B. (2014). iGC2: An architecture for micro gas chromatographs utilizing integrated bi-directional pumps and multi-stage preconcentrators. J. Micromech. Microeng..

[22] Qin Y., Gianchandani Y.B. An all electronic, fully microfabricated micro gas chromatograph. Proceedings of the IEEE International Conference on Solid-State Sensors, Actuators and Microsystems (Transducers).

[23] Qin Y., Gianchandani Y.B. (2016). A fully electronic microfabricated gas chromatograph with complementary capacitive detectors for indoor pollutants. Microsyst. Nanoeng..

[24] Azzouz I., Poulichet P., Pirro M., Tan W., Marty F., Capochichi-Gnambodoe M., Nefzaoui E., Boumechhour A., Cesar W., Angelesc D. Evaluation of Tenax thin films as adsorbent material in a micro-preconcentrator and its operation as a valve-less multiple injection system in micro-gas chromatography. Proceedings of the IEEE International Conference on Solid-State Sensors and Actuators (Transducers).

[25] Oh K.W., Ahn C.H. (2006). A review of microvalves. J. Micromech. Microeng..

[26] Robertson J.K., Wise K.D. (1998). A low pressure micromachined flow modulator. Sens. Actuators A Phys..

[27] Bosch D., Heimhofer B., Muck G., Seidel H., Thumser U., Welser W. (1993). A silicon microvalve with combined electromagnetic/electrostatic actuation. Sens. Actuators A Phys..

[28] Bae B., Han J., Masel R.I., Shannon M. (2007). A bidirectional electrostatic microvalve with microsecond switching performance. J. Microelectromech. Syst..

[29] Kim H., Najafi K. Electrostatic hydraulic three-way gas microvalve for high-pressure applications. Proceedings of the 12th International Conference on Miniaturized Systems for Chemistry and Life Sciences (µTAS).

[30] Wijngaart W.V.D., Ask H., Enoksson P., Stemme G. (2002). A high-stroke, high-pressure electrostatic actuator for valve applications. Sens. Actuators A Phys..

[31] Tice J.D., Desai A.V., Bassett T.A., Apblett C.A., Kenis J.A. (2014). Control of pressure-driven components in integrated micro fluidic devices using an on-chip electrostatic microvalve. RSC Adv..

[32] Esashi M., Shoji S., Nakano A. (1989). Normally closed valve microvalve and micropump fabricated on a silicon wafer. Sens. Actuators.

[33] Shoji S., Schoot B.V.D., Rooij N.D., Esashi M. Smallest dead volume microvalves for integrated chemical analyzing systems. Proceedings of the IEEE International Conference on Solid-State Sensors and Actuators (Transducers).

[34] Roberts D.C., Li H., Steyn J.L., Yaglioglu O., Spearing S.M., Schmidt M.A., Hagood N.W. (2003). A piezoelectric microvalve for compact high-frequency, high-differential pressure hydraulic micropumping systems. J. Microelectromech. Syst..

[35] Groen M.S., Brouwer D.M., Lӧtters J.C., Wiegerink R.J. (2015). Miniature proportional control valve with top mounted piezo bimorph actuator with millisecond response time. J. Micromech. Microeng..

[36] Lu H.-T., Qin Y., Gianchandani Y.B. A hybrid three-way valve for gas chromatography systems. Proceedings of the 2018 IEEE Sensors Conference.

[37] Rich C.A., Wise K.D. (2003). A High-Flow Thermopneumatic Microvalve with Improved Efficiency and Integrated State Sensing. J. Microelectromech. Syst..

[38] Potkay J.A., Wise K.D. (2012). A hybrid thermopneumatic and electrostatic microvalve with integrated position sensing. Micromachines.

[39] Shinozawa Y., Abe T., Kondo T. A proportional microvalve using a bi-stable magnetic actuator. Proceedings of the IEEE/ASME International Conference on Micro Electro Mechanical Systems (MEMS).

[40] Bӧhm S., Burger G.J., Korthorst M.T., Roseboom F. (2000). A micromachined silicon valve driven by a miniature bi-stable electro-magnet actuator. Sens. Actuators A Phys..

[41] Fu C., Rummler Z., Schomburg W. (2003). Magnetically driven micro ball valves fabricated by multilayer adhesive film bonding. J. Micromech. Microeng..

[42] Grover W.H., von Muhlen M.G., Manalis S.R. (2008). Teflon films for chemically-inert microfluidic valves and pumps. Lab Chip.

[43] Liao W., Zhao X., Lu H.-T., Qin Y., Gianchandani Y.B. Progressive cellular architecture in gas chromatograph for broad vapor sensing. Proceedings of the Chemical and Biological Defense Science & Technology (CBD S&T) Conference.

[44] Bruss H. (2007). Hydraulic resistance and compliance. Theoretical Microfluidics.

[45] Foley J.P., Dorsey J.G. (1983). Equations for calculation of chromatographic figures of merit for ideal and skewed peaks. Anal. Chem..

